# Losmapimod, an Oral Anti-p38 MAP Kinase, Demonstrates Anti-Neuropathic and Anti-Inflammatory Effects in Rat Acute Pain

**DOI:** 10.3390/jcm15114265

**Published:** 2026-05-31

**Authors:** Mickael Soued, Leila Hamdi, Mouna Ben Rehouma, Jean-Xavier Mazoit, Dan Benhamou

**Affiliations:** 1 Laboratory of Anesthesia, Inserm U 1195 Neuroprotection et Neuroregeneration, Paris-Saclay University, 94270 Le Kremlin-Bicêtre, France; 2 Department of Anesthesia, Antoine Béclère Hospital, APHP, Paris-Saclay University, 92140 Clamart, France; 3 Clinique les Martinets, Groupe Ramsay Santé, 92500 Rueil-Malmaison, France; 4 Department of Anesthesia and Intensive Care Medicine, Bichat Hospital, APHP, Paris Seine Saint Denis, 75018 Paris, France; 5 Department of Anesthesia and Intensive Care Medicine, Bicêtre Hospital, APHP, Paris-Saclay University, 94270 Le Kremlin-Bicêtre, France

**Keywords:** losmapimod, p38 MAPK, acute neuropathic pain, inflammation, rat

## Abstract

**Background:** The postoperative period is a large provider of acute and chronic pain which often combine pronociceptive, neuropathic and inflammatory components. As p38 MAPkinases are involved in pain response, this study aimed to evaluate the effect of losmapimod, an oral p38 MAPkinase inhibitor at the very first stage of acute neuropathic pain. Losmapimod efficacy was also compared with other well-known pain-relieving drugs and its dose response determined after inflammatory pain. **Methods:** The anti-neuropathic properties of losmapimod were evaluated after acute neuropathic pain (from day 0 to day 3 after sciatic nerve ligation) using thermal and mechanical stimulation. Losmapimod was also compared with gabapentin, their respective ED_50_ were determined, and their interaction was studied using an isobolographic approach. The anti-inflammatory characteristics of losmapimod were assessed from day 0 to day 5 after carrageenan injection in the rat hind paw and compared with those of ketoprofen, ketamine, and morphine using paw oedema volume. **Results:** Losmapimod provided a potent analgesic effect after acute neuropathic pain. The ED_50_ with their 95% confidence intervals of losmapimod and gabapentin were 10.5 (6.9–12.9) mg/kg and 18.2 (15.0–20.3) mg/kg, respectively. Their interaction was additive. Losmapimod also had a potent anti-inflammatory effect (ED_50_ was 34 (19.0–246) mg/kg) with significant reduction of paw oedema (similarly to ketoprofen). **Conclusions:** Losmapimod showed anti neuropathic properties at the very early stage of neuropathic pain and potent anti-inflammatory properties. As previous studies have highlighted the excellent tolerance of losmapimod in human populations, this drug seems promising for acute postoperative pain, which combines both acute neuropathic and inflammatory mechanisms.

## 1. Introduction

Currently, over 300 million surgical procedures are carried out each year [1]. Recent audits [2,3] highlight that over 80% of patients experience immediate postoperative pain, with 40% still experiencing moderate or severe pain [3]. Postoperative pain is often considered to be caused by an excess of nociception mainly driven by the inflammatory reaction, but an authentic acute neuropathic component could be present [4,5,6], even in the absence of obvious direct nerve damage [5]. This neuropathic character may represent a new target and early effective management could reduce the risk of chronicisation [4].

Gabapentin is effective in treating chronic neuropathic pain [7] and has been widely used in the preoperative setting for the last two decades. However, its use was not specifically intended to reduce acute neuropathic pain, but rather to reduce postoperative pain in general, in the hope that it would lower the risk of chronicisation. Today, its use in acute postoperative pain is widely debated, particularly given its limited efficacy in reducing usual pain scores and the risk of side effects [8,9]. To substantiate the potential efficacy of drugs in treating acute neuropathic pain, experimental studies are needed first, as clinical studies are not yet available in this situation.

Different pain stimuli, including neuropathic pain, activate p38 mitogen-activated protein kinase (MAPK) in the primary afferent neurons, as well as in spinal microglial and astrocytic cells [10]. This activation generates a chronic spinal pro-inflammatory state and is the first step leading to chronic pain [11]. p38 inhibitors such as SB203580 reduce allodynia and hyperalgesia following inflammatory stimulation; however intrathecal administration limits their use [12]. Losmapimod, an orally available p38 inhibitor, has recently been successfully tested following carrageenan injection and plantar incision in rats [13]. Moreover, given the excellent tolerance of losmapimod reported in humans [14,15,16,17], its evaluation in the context of acute neuropathic and inflammatory pain seems warranted in order to complete the study of all of the characteristics of acute postoperative pain (from the excess of nociception already investigated [13] to the neuropathic and inflammatory components).

This study therefore had several aims: (1) to evaluate the efficacy of losmapimod in the earliest period of neuropathic pain following partial sciatic nerve ligation in rats; (2) to determine the effective dose of losmapimod and gabapentin for 50% of the experimental population (ED_50_), and then to study their combination; (3) to measure the anti-inflammatory potency of losmapimod following carrageenan injection; and (4) to describe the related dose–response curve.

## 2. Materials and Methods

All experiments were approved by the Animal Ethics Committee (CEEA 26, Paris Sud, N°4889/2016041110429969 and 15570/2018101210036429, approved on 6 June 2016 and 15 December 2018, respectively) and were conducted in accordance with ARRIVE guidelines. Male Sprague–Dawley rats (Janvier Labs, 53940 Le-Genest-Saint-Isle, France), which are known to be docile and easy to handle, were used for this behavioural study. The rats were housed in groups of three per cage at 22 °C (±2 °C), with free access to food and water, under a 12 h light-dark cycle, exclusively for this study. All procedures were performed under general isoflurane anaesthesia. After testing, the rats were euthanised by CO_2_ inhalation gradient.

### 2.1. Pain-Producing Procedures and Drugs Administered

The partial sciatic ligation (PSL) was performed on Day 0 (D0), as previously described by Kim and Chung [18]. Briefly, the rat was placed in a prone position, and an incision was performed medially between fourth lumbar and second sacral vertebrae. Para spinae muscles were gently dissected, and the fifth left lumbar root was tightly ligated just distal to the dorsal root ganglia. The different anatomical planes were sutured, and topical antibiotics were applied to the skin.

Carrageenan injection (100 µL of carrageenan 3% (Sigma, Saint-Quentin-Fallavier, France) diluted in saline (0.9% NaCl) was performed subcutaneously to the left hind paw on D0.

All of the different drugs were administered daily, once a day. Gabapentin (Biogaran, Colombes, France), losmapimod (Euromedex, Souffelweyersheim, France), and paracetamol (Panpharma, Luitré-Dompierre, France) were administered orally, via a gavage cannula. The administration was considered successful if the rat did not cough or vomit. Ketoprofen (Medisol, Puteaux, France), ketamine (Panpharma, Luitré-Dompierre, France), and morphine (CDM Lavoisier, Paris, France) were injected subcutaneously into the abdominal wall.

Gabapentin and losmapimod efficacies were evaluated after PSL. Losmapimod, paracetamol, ketoprofen, ketamine, and morphine were administered following carrageenan injection.

### 2.2. Evaluation of Analgesic Effects of Losmapimod After PSL

To reduce rat stress and thus facilitate further cooperation, the rats were acclimatised for 10 days prior to behavioural evaluation. During this period, von Frey filaments (for assessing mechanical stimulation) and a Hargreaves heat lamp (for assessing thermal stimulation) were applied until stable baseline values were obtained. For von Frey stimulation, the filaments were applied 10 times in ascending order of stiffness. Filament stiffness was incremented progressively after a positive test result (defined as at least six paw withdrawals per ten applications). The Hargreaves test was performed using a radiant heat source focused under the ipsilateral paw. A 20 s cut-off was applied to avoid tissue damage.

Two groups were compared: PSL alone and PSL with losmapimod (L + PSL). Tests were performed once a day from D0 to D3. Losmapimod was administered daily from D0 to D2. The anti-neuropathic properties of losmapimod were tested after PSL at a dose of 30 mg/kg, which corresponds to the ED90 following carrageenan injection [13].

### 2.3. Investigation of Losmapimod and Gabapentin ED_50_s and Analysis of Their Interaction

The doses of losmapimod and gabapentin required to induce mechanical analgesia in 50% of rats (ED_50_) were estimated using the “up and down” allocation method followed by central isotonic regression with the R package cir (version 4.3.0) (R core team 2018, Vienna, Austria) [19]. Briefly, the first von Frey filament applied to the rat paw corresponded to the threshold value (D0) before sciatic ligation. An initial dose was administered to the animal, and subsequent doses were given according to the following rule. The initial doses were 10 mg/kg for losmapimod and 20 mg/kg for gabapentin, with dose intervals of 3 mg for both drugs. If the animal responded positively, the dose administered to the next animal was decreased by one interval. Conversely, if the animal responded negatively, the dose administered to the next animal was increased by one interval.

Once the ED_50_ for each drug had been determined, the drugs were administered in combination at a ratio determined by the ratio of the ED_50_s, and the ED_50_ for the combination was estimated for isobolographic analysis [17]. In addition, the 84% and 95% confidence intervals (CI84 and CI95) for the ED_50_s were also estimated for statistical comparison and graphical representation. The combination of the two molecules was considered.

- additive if the CI84s of the ED_50_s overlapped;
- synergistic if the ED_50_ of the combination and its CI84 were below the line joining the CI84 lower limit of the two ED_50_s;
- infra-additive if the ED_50_ of the combination and its CI84 were above the line joining the CI84 upper limit of the two ED_50_s [20].

### 2.4. Evaluation of Anti-Inflammatory Effects After Carrageenan Injection

In this sub-study, paw oedema induced by carrageenan injection was used to measure the intensity of local inflammation. In this aim, acclimatisation was also performed but without the application of von Frey hairs or the Hargreaves lamp. The rat paw was immersed in a plethysmometer which measures paw volume by calculating the volume of displaced water after immersion. Paw volume was measured once a day during acclimatisation and from D0 to D5.

Seven groups were compared: carrageenan or losmapimod alone and carrageenan combined with either losmapimod, morphine, paracetamol, ketoprofen, or ketamine. Losmapimod was administered at 12 mg/kg because this was the only previously described dose in an inflammatory context [21]. Paracetamol, ketamine, ketoprofen, and morphine were administered at 500 mg/kg [22], 20 mg/kg [23], 5 mg/kg [24], and 3 mg/kg [25], respectively. A 3% carrageenan injection was performed on D0 and studied drugs were administered daily from D0 to D2, while paw volume was measured daily from D0 to D5.

The methods used to calculate the dose–response curve were similar to those used in a previous study [13]. In brief, the previously described effective dose in rats is 12 mg/kg [21]. We used five doses (0–2–4–12–50 mg/kg) in an attempt to cover the entire curve, as was previously performed for analgesic properties [13].

The effect of losmapimod (E) was calculated according to the maximum effect model (Emax) (E = E0 – Emax × D/(ED_50_ + D)), where E0 and Emax represent the theoretical minimum and maximum effects respectively, D is the administered dose and ED_50_ is the dose inducing 50% of the maximum effect (Emax − E0/2). We used the NONlinear Mixed Effects Modelling (NONMEM) program version VI (ICON Clinical Research LLC, Dublin, Ireland).

The 95% confidence interval (95% CI) of the parameters was calculated using log-likelihood profiling.

All procedures, studied groups, behavioural testing and drug administrations are summarised in Table 1.

### 2.5. Statistical Analysis

Statistical analyses were performed using the R software (R core team version 4.4.1 (2024), R foundation for Statistical Computing, Vienna, Austria). The minimum number of rats required for each group (excluding the up-and-down and dose–response sub-studies) was calculated based on 80% power and a 5% α risk (bilateral, corrected using the Bonferroni correction for the number of planned comparisons). As most of the variables (e.g., mechanical and thermal thresholds and volume) were not normally distributed (as indicated by Shapiro–Wilk tests and qq plots), the non-parametric NparLD package was used, with time as the repeated measure and group as the factor. Baseline values and post-hoc comparisons were analysed using the Kruskal–Wallis or Mann–Whitney test, respectively, with Holm’s correction as appropriate. Results are expressed as median [interquartile range]. A *p*-value of less than 0.05 was considered statistically significant.

Rats were randomly assigned to each group.

The statistical analysis was performed by MS and JXM, who were blinded to the group allocation and people performing procedures were blinded as drugs were prepared by another team member.

### 2.6. Side Effects

Side effects were investigated and recorded. Rats were also monitored for signs of distress such as weight loss exceeding 10% of the initial weight, hair loss and serious behaviour disturbances. If any of these were present, the rats were euthanised.

## 3. Results

A total of 172 rats were included in this study.

### 3.1. Anti-Neuropathic Properties of Losmapimod After PSL on Left Hind Paw (Ipsilateral) Withdrawal Thresholds (Figure 1)

Ten rats were included in each of the PSL and L + PSL groups. There was no difference in baseline values (at D0) between the two groups (*p* = 0.17 and *p* = 0.07 for mechanical and thermal stimulation, respectively). Following PSL, paw withdrawal thresholds were significantly reduced in response to both mechanical and thermal stimulation (*p* < 0.0001 for both). Losmapimod administration significantly increased the paw withdrawal threshold (*p* < 0.01 for mechanical stimulation and *p* = 0.01 for thermal stimulation). Paw withdrawal thresholds were significantly different between L + PSL and PSL groups (*p* < 0.01 for both stimulations).

**Figure 1:**
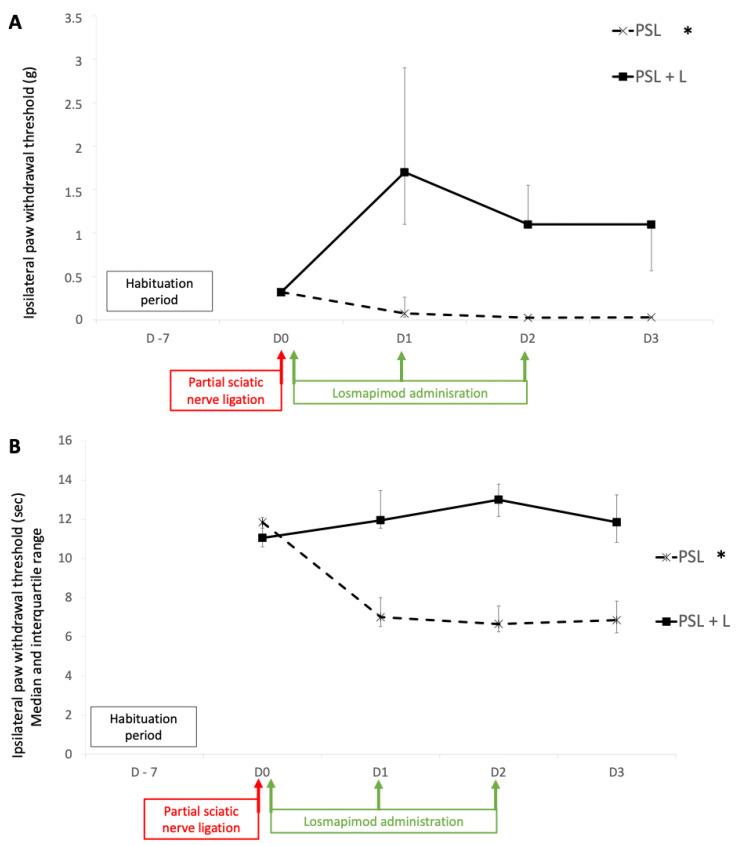
Left paw withdrawal thresholds, in grams and seconds, after respective mechanical (**A**) and thermal (**B**) stimulation as a function of time. Data are expressed as median and interquartile range. Ten rats per group were included. *: *p* < 0.01 between groups. PSL: partial sciatic nerve ligation. PSL + L: partial sciatic nerve ligation + losmapimod.

### 3.2. ED_50_ of Losmapimod, Gabapentin and Their Interaction (Figure 2 and Figure 3)

**Figure 2:**
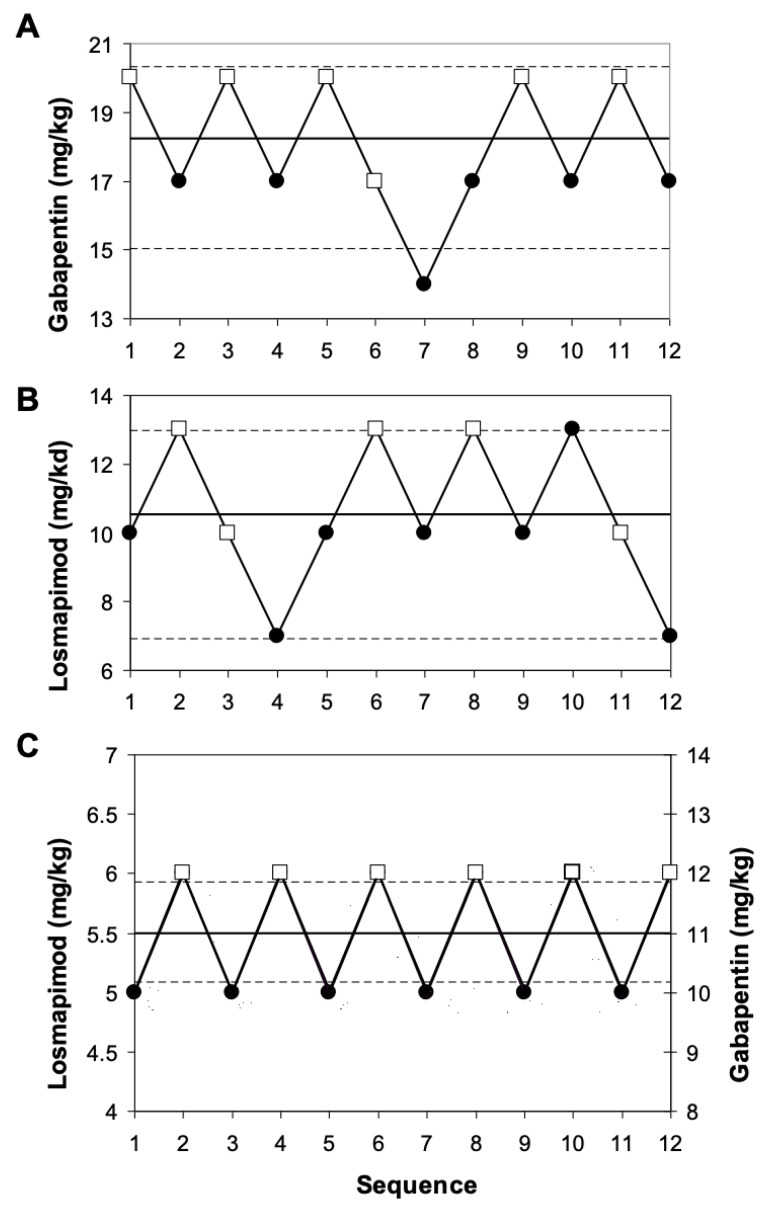
Sequences of dosing for the three groups of 12 rats after sciatic nerve ligation in rats: losmapimod (**A**), gabapentin (**B**), and the combination of the two (**C**). Solid circles correspond to a positive response (return of the withdrawal threshold to the threshold value) and empty squares to a negative response (absence of return of the withdrawal threshold to the threshold value). Scheme 50 and dotted lines to 95% CI.

**Figure 3:**
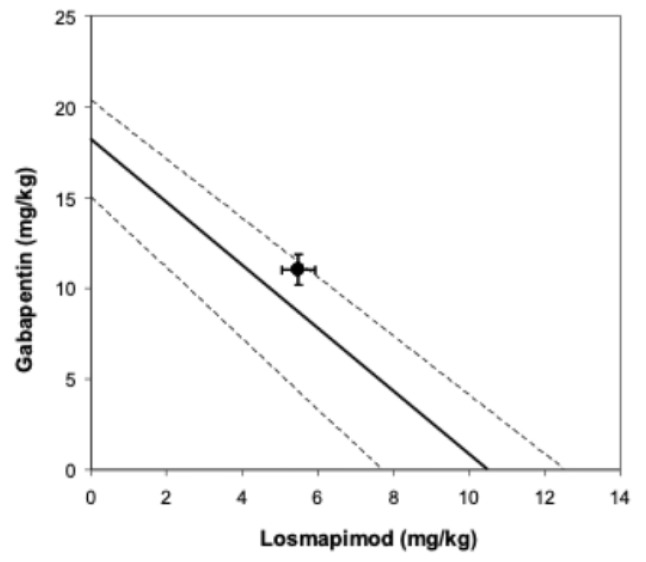
Isobolographic representation of ED_50_ of losmapimod and gabapentin and their combination after sciatic nerve ligation in rats. The solid line joining the ED_50_ of each molecule on the abscissa and ordinate represents the isobole. This corresponds to the pure additivity of the molecules. The dotted lines join the CI84 bounds of the ED_50_ confidence intervals of the two molecules. The ED_50_ of their combination is represented by the solid circle with its 84% confidence interval.

Data obtained at D1 were used, as the greatest effect of losmapimod was observed at this time-point following mechanical stimulation. Three groups of 12 rats were studied after LSP (losmapimod, gabapentin and the combination). The ED_50_s with their 95% CIs were 10.5 mg/kg (6.9–12.9) for losmapimod and 18.2 mg/kg (15.0–20.3) for gabapentin (Figure 2A,B). The ED_50_s, with their 84%CI, were 10.5 mg/kg (7.7–12.5) and 18.2 mg/kg (16.9–19.4), respectively. For the losmapimod–gabapentin combination, the ED_50_s, with their 95% CIs, were 5.5 mg/kg (5.1–5.9) and 11.0 mg/kg (10.2–11.8) for losmapimod and gabapentin, respectively (Figure 2C). Their ED_50_s with 84%CI were 5.5 (5.2–5.8) and 11.0 (10.5–11.5) (Figure 3). The interaction between the two molecules was not significantly different from additivity.

### 3.3. Anti-Inflammatory Effect of Losmapimod (Figure 4)

Each of the seven groups included twelve rats. Losmapimod alone did not modify paw volume (*p* = 0.22 over time). Carrageenan significantly increased the volume of the rat paw (*p* < 0.01 over time), and losmapimod administration reduced oedema (*p* < 0.01, losmapimod + carrageenan group vs. carrageenan group). Losmapimod reduced only partially the paw oedema (*p* < 0.01, losmapimod group vs. losmapimod + carrageenan group). Losmapimod and ketoprofen produced a similar anti-inflammatory effect (*p* = 0.59), which was superior to the effect produced by ketamine, morphine, and paracetamol (*p* < 0.01 vs. the losmapimod + carrageenan group).

**Figure 4:**
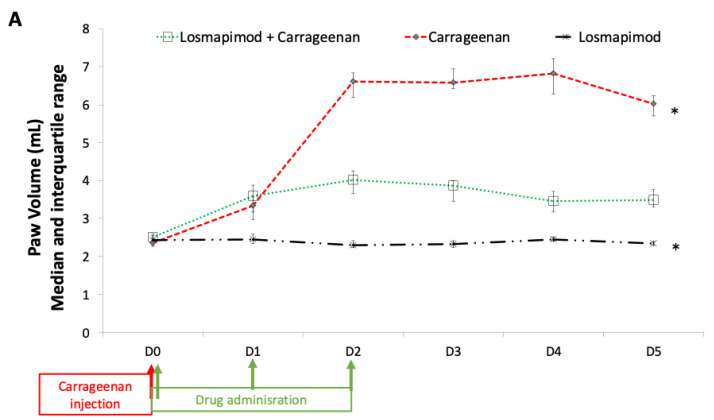

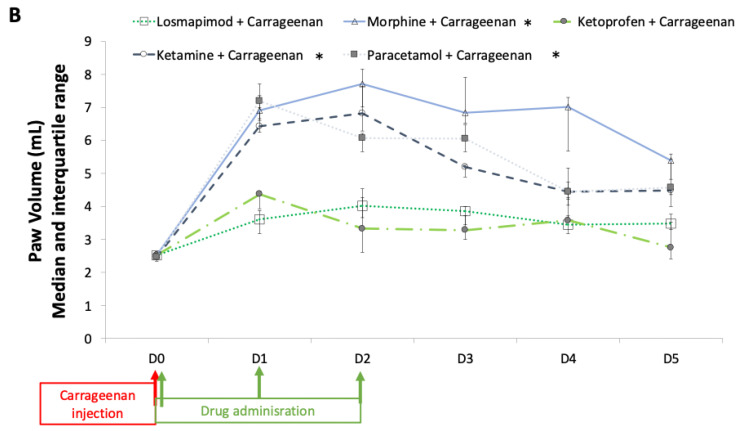
(**A**) Volume of the rat left paw (expressed in ml) as a function of time, in groups receiving carrageenan alone, losmapimod alone, and losmapimod after carrageenan injection. (**B**) After carrageenan injection, the effect of losmapimod is compared with that of morphine, ketamine, ketoprofen, and paracetamol. Data are expressed as medians and interquartile ranges. Twelve rats per group were included. *: *p* < 0.01 compared with losmapimod + carrageenan group.

### 3.4. Dose–Response Study in the Carrageenan Model (Figure 5)

Thirty-two rats were included. Paw volumes obtained on D1 were used to plot the dose/response curve, i.e., at the time of the maximum losmapimod effect. The dose of 12 mg/kg corresponded to an anti-inflammatory ED 26. The ED_50_ with its 95%CI was 34 (19.0–246) mg/kg while E0 and Emax were 4.5 mL and 1.6 mL, respectively.

**Figure 5:**
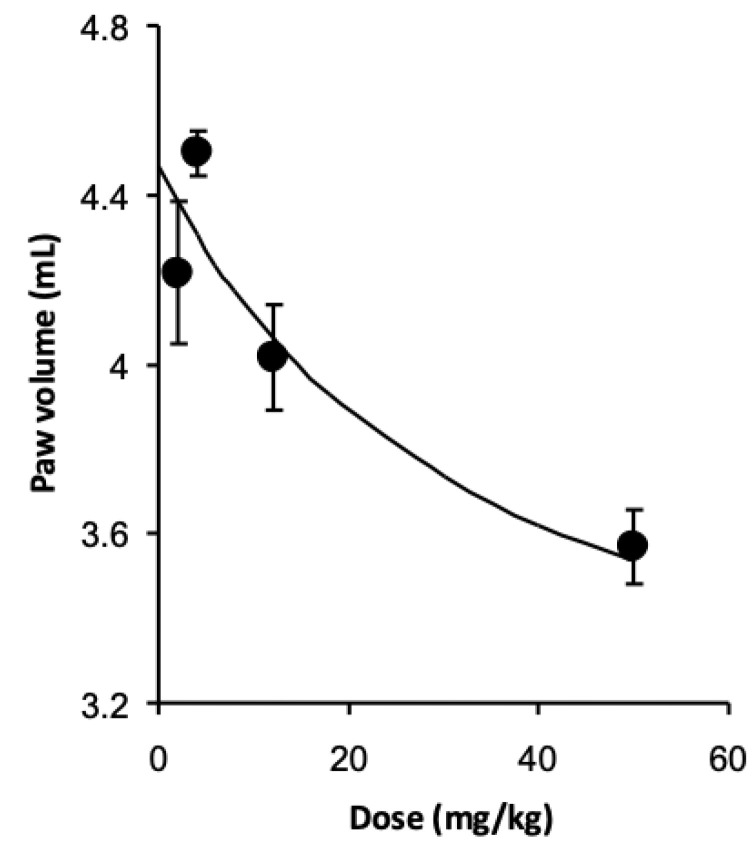
Dose–response study showing the effect of losmapimod on paw volume (expressed in mL) after carrageenan injection. Each point represents a dose administered (2, 4, 12 and 50 mg/kg). The y intercept corresponds to the 0 mg/kg dose. Overall, thirty-two rats were included in this sub-study.

### 3.5. Side Effects

No serious side-effects were noted during the entire study.

## 4. Discussion

This study demonstrated that (1) losmapimod provides potent analgesia in a rat model of acute neuropathic pain (partial sciatic nerve ligation [PSL]) with an ED_50_ of 10.5 mg/kg; (2) the interaction between losmapimod and gabapentin was additive in this model; and (3) after carrageenan injection in the rat paw, losmapimod exhibited potent anti-inflammatory properties, comparable to those of ketoprofen, with and ED_50_ of 34 mg/kg.

The marked analgesic effect in acute neuropathic pain is consistent with that obtained with other p38 inhibitor in rats [12,26], although SB203580, which was used in earlier studies, only partially reversed allodynia and hyperalgesia induced by peripheral nerve injury. Although the neuropathic component may be present from the very first postoperative hours [6], it does not currently represent a therapeutic objective in clinical practice. If painful stimuli are not managed from the outset, they induce neuronal and microglial activation [13], corresponding to the first stage of pain chronicisation [11,27,28]. Our study shows a potentially clinically relevant effect of losmapimod over the study period from D0 to D3. This is an important finding, as in experimental studies of neuropathic pain, the first point of measurement was either on D3 [26,29] or did not begin until 1 month or more after ligation. This creates confusion as to the type of pain explored (acute, subacute or chronic neuropathic pain) and hence the interpretation of results.

To date, losmapimod has been evaluated in the context of (chronic) neuropathic pain in humans [15,30]. The results showed no efficacy, leading to the conclusion that p38 inhibitors are not useful in the treatment of pain [31,32,33]. For example, in a study by Ostenfeld et al., adult patients suffering from neuropathic pain for months or even years were included and pain pathways may have become well established and difficult to reverse [15].

In our study, the ED50 dose of losmapimod required to alleviate neuropathic pain following administration of PSL was found to be 10.5 mg/kg. A gross estimation of the equivalent dose in humans would be around 1.7 mg/kg [34] corresponding to a daily dose around 100 mg in a human adult weighing 60 kg. Most studies have used a daily dose of 15 mg, especially the two studies by Ostenfeld et al. [15,30] on chronic neuropathic pain which found the absence of a pain-relieving effect. In the discussion of these two articles [15,30], it was suggested that the absence of analgesia might be the consequence of a dose that is too low. This hypothesis was refuted on the basis of a hypothetical pharmacokinetic model suggesting that the dose used was sufficient to achieve the expected pharmacodynamic effects (apart from the limitation related to the tolerance threshold). The dose limitation was linked to the fact that unpublished data from the primary holder of losmapimod (GSK) had shown, in unpublished documents, that the maximum tolerated dose was 15 mg per day. However, other studies have used a dose of 30 mg per day [14,35] (<0.5 mg/kg) without any side effects. In a study focusing on the effect of losmapimod on the QT interval, the authors describe another unpublished study in which a dose of 60 mg was used without adverse effects in volunteers [36].

These data suggest that the dose used by Ostenfeld et al. was in fact too low. Our results confirm that a dose equivalent to 1.7 mg/kg was well tolerated in animals and produced a potent analgesic effect. Although acute neuropathic pain is a different condition from chronic neuropathic pain, it is interesting to note that Ostenfeld et al. [15] observed that a dose of up to 10 mg/kg was effective in a chronic constriction lesion model in rats, therefore corroborating our own data. Overall, this could lead to a new research programme in humans involving increasingly higher doses.

The ED_50_ of losmapimod after PSL was 10.5 mg/kg, which should be compared with the ED_50_ of losmapimod after carrageenan injection, i.e., 6.8 mg/kg [13]. These very similar values were obtained with different pain models. These results suggest that the different types of pain act, at least in part, through common cellular and molecular mechanisms, resulting in a similar response to losmapimod.

**Figure 6:**
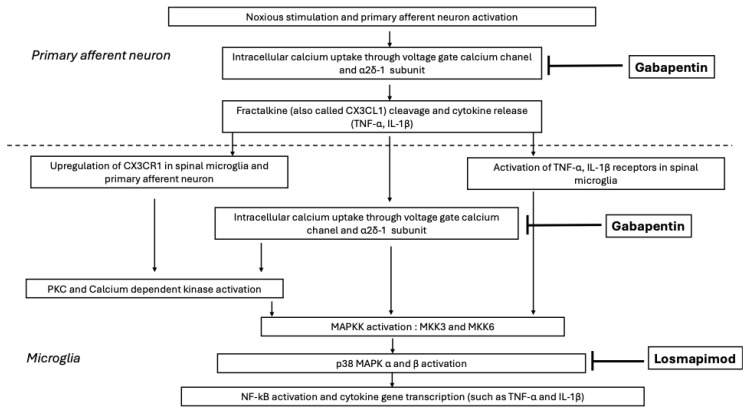
Representation of the potential interactions between losmapimod and gabapentin pathways [39,46,47].

We found an ED_50_ for gabapentin of 18.2 mg/kg and several other studies have also found an ED_50_ around 20 mg/kg following either sciatic nerve ligation [37], or carrageenan injection [38]. These results reinforce the validity of our study. The additive interaction between gabapentin and losmapimod suggests common cellular and molecular mechanisms. Indeed, several mechanistic studies have established a link between gabapentin and MAPK signalling pathways, including p38. In an inflammatory arthritis rat model, gabapentin inhibited CX3CL1 and the subsequent p38-mediated inflammatory cascade, thereby reducing microglial activation, through its action on the alpha-2/delta-1 subunit of the calcium channel, which is highly expressed in primary afferent neurons and the spinal cord [39]. Gabapentinoids also reduce pro-inflammatory cytokine production by inhibiting NFkappaB and p38 [40]. This interrupts the glutamate signalling pathway induced by voltage-gated calcium channels inhibition [39]. The addition of losmapimod to gabapentin in postoperative neuropathic and/or inflammatory situations could thus improve gabapentin efficacy, reducing the required dose and the undesirable dose-dependent [41] effects [42] (Figure 6).

Inflammation is an integral part of acute pain and the central role of p38 in the pro-inflammatory cascade is known for many years. Numerous studies have demonstrated that activation of p38 by an external stimulus induces the phosphorylation of transcription factors, ultimately leading to the release of pro-inflammatory cytokines [40]. Therapeutic inhibition targeting the p38 pathway has also been shown to reduce inflammation in various pathological contexts [40]. To our knowledge, this is the first study to address an objectively measured inflammation-induced sign (i.e., paw oedema) and draw a parallel between the efficacy of a p38 inhibitor and that of a traditional anti-inflammatory drug (i.e., ketoprofen). However, losmapimod may have significant advantages over ketoprofen. Indeed, ketoprofen (like other non-steroidal anti-inflammatory drugs) has many undesirable side effects which limit its use. Combining these two molecules could reduce these side effects by lowering the required dosage of each drug.

Although ketamine, paracetamol, and morphine reduce pain, they do not reduce the inflammatory oedema generated by carrageenan. These molecules have no anti-inflammatory effect, which confirms that our study has indeed focused on inflammation and that our endpoint allows us to dissociate between the pain induced by inflammation and other types of pain.

Our dose–response analysis revealed that the anti-nociceptive effect is stronger than the anti-inflammatory one, as the 12 mg/kg dose produced an approximately ED_70_ analgesic effect [13], whereas only an ED_26_ anti-inflammatory effect was observed.

Moreover, despite the significant effect on paw oedema, even at the highest doses, complete regression of the oedema was not observed. As a number of human studies have demonstrated the tolerability of losmapimod [14,15,16], it appears feasible to safely increase the dose [17], as discussed above.

To explain the difference between anti-neuropathic and anti-inflammatory effects, we can assume that some of the oedema is painless. This could explain why the clinical anti-inflammatory effect appears to be weaker than the analgesic effect, with painful symptoms disappearing before the oedema has fully regressed.

From a mechanistic perspective, it is also possible that the analgesic effect of losmapimod is primarily spinal (as previously reported for a dose of 12 mg/kg, which is sufficient to inhibit both the primary afferent neurons and the central microglial activation [13]), whereas its anti-inflammatory effect, particularly via cyclooxygenase inhibition, may be peripheral and require a higher dosage.

The main limitation of our study concerns the murine model itself, which raises question about its validity. While our team has extensive experience in rat behavioural evaluation [13,29,43,44], and our results concur with those previously obtained with two pain models [13], several authors consider that transferring experimental results from rats to humans is too much of a risk [31], as many promising molecules that were effective in animals were not effective in humans. A related concern is that human studies found no benefit of losmapimod in patients with chronic lumbosacral radiculopathy [15], whereas we obtained effective analgesia in murine models of acute pain. This apparent difference in effect between acute and chronic pain may be due to the different pain pathways, and is similar to what has recently been found with suzetrigine, which has been found to be effective in postoperative pain but not in patients with chronic lumbo-sacral neuropathy [45]. This underscores the importance of developing new non-opioid drugs especially for use in the postoperative context.

Lastly, our study suggests that gabapentin and losmapimod have some common therapeutic targets. However, this is based on pharmacological interaction data, and we have not conducted any mechanistic analysis at molecular and/or cellular level. Such analyses could be useful in the future, in order to authenticate the targets of losmapimod and gabapentin, as well as the effect of their interaction, on the entire inflammatory chain.

In conclusion, this study demonstrated the efficacy of losmapimod in treating acute neuropathic pain in a murine model, as well as its additive effects when used alongside gabapentin. Losmapimod also has a marked anti-inflammatory effect and targets several important steps in the acute pain process. These data suggest that losmapimod may be evaluated in future human acute pain research trials.

## Figures and Tables

**Table 1:** Description of experimental procedures, time points of drug administrations and behavioural evaluations.

	Groups, Drug Administered	10 Days of Acclimatisation	Procedural Stimulation	Evaluation	D0	D1	D2	D3	D4	D5
Neuropathic pain	Losmapimod vs. Control *n* = 12 per group	X	Partial sciatic nerve ligation	Hargreaves lamp and von Frey hairs	D + E	D + E	D + E	E		
	Losmapimod, Gabapentin ED 50 and interaction *n* = 36 rats in total					D + E				
Inflammation	Carrageenan and losmapimod alone. Carrageenan in combination with paracetamol, ketoprofen, morphine, ketamine or losmapimod *n* = 12 per group	X	Carrageenan injection	Plethysmometry	D + E	D + E	D + E	E	E	E
	Losmapimod dose response *n* = 32 rats in total					D + E				

D*n* = day after injury; D = drug administration; E = behavioural evaluation.

## Data Availability

This article includes only data generated during the study, which may be made available upon reasonable request.
